# Optimum polygenic profile to resist exertional rhabdomyolysis during a marathon

**DOI:** 10.1371/journal.pone.0172965

**Published:** 2017-03-03

**Authors:** Juan Del Coso, Marjorie Valero, Juan José Salinero, Beatriz Lara, César Gallo-Salazar, Francisco Areces

**Affiliations:** Exercise Physiology Laboratory, Camilo José Cela University, Madrid, Spain; King Abdullah International Medical Research Center, SAUDI ARABIA

## Abstract

**Purpose:**

Exertional rhabdomyolysis can occur in individuals performing various types of exercise but it is unclear why some individuals develop this condition while others do not. Previous investigations have determined the role of several single nucleotide polymorphisms (SNPs) to explain inter-individual variability of serum creatine kinase (CK) concentrations after exertional muscle damage. However, there has been no research about the interrelationship among these SNPs. The purpose of this investigation was to analyze seven SNPs that are candidates for explaining individual variations of CK response after a marathon competition (ACE = 287bp Ins/Del, ACTN3 = p.R577X, CKMM = NcoI, IGF2 = C13790G, IL6 = 174G>C, MLCK = C37885A, TNFα = 308G>A).

**Methods:**

Using Williams and Folland’s model, we determined the total genotype score from the accumulated combination of these seven SNPs for marathoners with a low CK response (n = 36; serum CK <400 U·L^-1^) *vs*. marathoners with a high CK response (n = 31; serum CK ≥400 U·L^-1^).

**Results:**

At the end of the race, low CK responders had lower serum CK (290±65 *vs*. 733±405 U·L^-1^; *P*<0.01) and myoglobin concentrations (443±328 *vs*. 1009±971 ng·mL^-1^, *P*<0.01) than high CK responders. Although the groups were similar in age, anthropometric characteristics, running experience and training habits, total genotype score was higher in low CK responders than in high CK responders (5.2±1.4 vs. 4.4±1.7 point, *P* = 0.02).

**Conclusion:**

Marathoners with a lower CK response after the race had a more favorable polygenic profile than runners with high serum CK concentrations. This might suggest a significant role of genetic polymorphisms in the levels of exertional muscle damage and rhabdomyolysis. Yet other SNPs, in addition to exercise training, might also play a role in the values of CK after damaging exercise.

## Introduction

Exertional rhabdomyolysis is a condition in which strenuous exercise or vigorous muscle activity results in ultrastructural damage to the skeletal muscle. This exercise-induced muscle damage is characterized by a reduced capacity to generate force and a reduced range of motion, delayed-onset muscle soreness and increases of intramuscular proteins in the systemic circulation [1] that might lead to myoglobinuria [2]. Although exertional rhabdomyolysis is resolved without serious medical complications in most physical activity and sport situations, this condition can be fatal due to myoglobin-induced renal failure and/or cardiac arrhythmia, especially in hot environments [3].

Exertional rhabdomyolysis can occur in individuals performing different types of exercise but it is unclear why some individuals develop exertional rhabdomyolysis when participating in comparable levels of physical exercise when other individuals do not develop this condition [4]. In research, the most widely used marker to evaluate the level of exertional rhabdomyolysis is the serum concentration of the enzyme creatine kinase (CK; [5, 6]). Previous investigations have determined a great inter-individual variability of serum CK concentrations after typical sport situations such as the marathon [7], triathlon [8], or after other types of exercise [6, 9]. This suggests that the CK inter-individual response is one of the causes for the development of exertional rhabdomyolysis in young and otherwise healthy individuals. Interestingly, several research groups have coincided in proposing that genetics might play a fundamental role in the inter-individual differences found in CK response after exercise and hence their vulnerability to exertional rhabdomyolysis. The identification of genotypes related to a higher probability of developing high levels of muscle damage during exercise could be very useful to aid in the prevention of exertional rhabdomyolysis in athletes, military personnel and civilians [10].

Based on this hypothesis, several genes and single nucleotide polymorphisms (SNP) have been related to typical signs of exertional rhabdomyolysis, especially the CK response. The angiotensin I-converting enzyme (ACE) insertion (I)/deletion (D) polymorphism has been one of the most targeted genes because of the solid association of the ACE II homozygosity with enhanced endurance performance [11]. However, the ACE II genotype might also increase the risk for developing exertional rhabdomyolysis because this genotype has been related to a higher CK response after eccentric exercise of the elbow [12], although this was not confirmed after a stepping exercise [5]. The α-actinin skeletal muscle isoform 3 (ACTN3) gene and its R577X SNP has been also related to exercise performance [13] because the 577XX null genotype leads to the production of a non-functional α-actinin-3 in type II fibers [14]. Although XX homozygotes for this SNP have an increased activity of mitochondrial enzymes involved in aerobic metabolism and a reduced activity of enzymes involved in anaerobic metabolism to compensate for the absence of α-actinin-3 [15], it is likely that these metabolic adaptations do not compensate the mechanical consequence derived from the lack of this protein. In fact, the majority of investigations indicate that XX homozygotes present greater signs of exertional rhabdomyolysis than their RR counterparts [10, 14, 16, 17]. The muscle type subunit of the creatine kinase enzyme (CKMM) and its encoding gene have also been related to the capacity of skeletal muscle to resist strenuous exercise due to its main role in energy production in the skeletal muscle. Specifically, AA homozygotes for the CKMM NcoI polymorphism had a six-fold higher risk of being a high CK responder after a stepping exercise [5], although others have found that GG homozygotes were 3.1 times more likely to experience exertional rhabdomyolysis than carriers of the A-allele in patients seeking medical attention for this condition [10].

The level of exertional rhabdomyolysis can also be influenced by the insulin-like growth factor (IGF) system and several of its IGF-binding proteins, such as IGF-II because of its role in modulating satellite cell activation and differentiation. To date, only one investigation has determined that several IGF2 SNPs can be related to muscle damage, specifically the C13790G, because GG homozygotes presented greater force loss, soreness and CK response than CC counterparts after eccentric exercise of the elbow [18]. The functional −174 G>C SNP of the interleukin-6 (IL-6) gene has also been linked to exertional muscle damage because C-allele carriers of this SNP presented higher CK values following eccentric elbow muscle contractions compared with GG homozygotes [19, 20]. The myosin light chain kinase (MLCK) phosphorylates the regulatory light chains, resulting in an increased number of force-generating cross-bridges [21]. The C37885A SNP of the MLCK gene and its influence on muscle damage has been studied with contradictory results. While it has been found that A-allele carriers for this MLCK SNP have greater muscle strength loss and increased plasma CK following eccentric contractions of the elbow flexor muscles [22], others have found that CA heterozygotes have fewer signs of muscle damage after a marathon than CC homozygotes [23]. Finally, tumor necrosis factor (TNFα) is a pro-inflammatory cytokine associated with the up-regulation of catabolic pathways and suppression of protein synthesis in skeletal muscle [24]. The TNF −308 (G>A) SNP has been related to exercise muscle damage because GG homozygotes presented higher values of CK than GA heterozygotes after eccentric exercise of the elbow [19, 20].

Most of the above-mentioned research has investigated the effect of genetics on exertional rhabdomyolysis/CK response to exercise by targeting one or two candidate genes and by using exercise protocols barely applicable to any real sport discipline (mostly eccentric exercise of the elbow). However, there is no information about the interrelationship among these genes and SNPs to resist CK response and thus the likelihood of developing exertional rhabdomyolysis during a real sport situation. Thus, the aim of this investigation was to determine the summed influence of seven candidate genes (ACE; ACTN3, CKMM, IGF2, IL6, MLCK, TNFα) on the levels of serum CK concentration and other signs of exercise-induced muscle damage attained during a real marathon competition. We have chosen a real marathon competition because this type of exercise is one of the most habitual scenarios for developing rhabdomyolysis [4, 25]. The hypothesis was that those participants with the best combination of genotypes would have lower levels of exercise-induced muscle damage.

## Methods

### Subjects

Sixty-seven healthy and experienced marathon runners (all Spanish Caucasians) volunteered to participate in this investigation. Potential participants were contacted from a group of runners that had participated in previous investigations or they were enrolled at the registration desk of the competition. Inclusion criteria were as follows: age between 18 and 65 years, being free of any history of muscle, cardiac or kidney disorders, participating in the marathon at maximal intensity, and having running experience of at least 3 years. Exclusion criteria were as follows: taking medications or nutritional supplements during the two weeks prior to competing or having suffered a bone or musculoskeletal injury in the 3 months before the competition. A pre-participation medical examination ensured the suitability of all participants to take part in the research protocols. Participants also completed a questionnaire about previous endurance running training, running experience and best race time in the marathon. Age, main morphological and physical characteristics and running training habits and experience are depicted in Table 1. The single influence of the SNPs associated to the MLCK gene [23] and the ACTN3 gene [26] on exercise-induced muscle damage in these same participants has been previously presented elsewhere.

**Table 1:** Age, anthropometric characteristics, best race time in the marathon, running experience, and training status of runners with low and high CK responses after a marathon. Data are mean ± SD.

Variable	Low CK responders	High CK responders	Δ	ES	P value
N	36	31	-	-	-
Men/women (%)	86.1/13.9	93.5/6.5	-	-	0.32
Age (yr)	43.0 ± 8.1	41.4 ± 9.4	-3.7%	0.20	0.47
Body mass (kg)	72.3 ± 11.7	74.1 ± 9.7	2.4%	0.15	0.54
Body height (cm)	174 ± 9	174 ± 6	0.1%	0.02	0.94
Best race time in the marathon (min)	217 ± 34	212 ± 32	-2.3%	0.15	0.58
Completed marathons (number)	8 ± 10	9 ± 10	14.1%	0.11	0.71
Running experience (yr)	10.1 ± 5.8	13.1 ± 11.6	28.8%	0.50	0.20
Average training distance /week (km)	62.8 ± 23.7	55.8 ± 23.6	-11.1%	0.29	0.25
Training sessions /week (number)	4.4 ± 1.0	4.6 ± 1.1	3.8	0.17	0.51

### Ethics statement

Once the inclusion/exclusion criteria were fulfilled, each participant was informed verbally and in writing of the benefits and hazards associated with the research protocols and signed an informed consent document. The study was approved by the Ethics Committee for Research of the Camilo Jose Cela University (Madrid). All the research protocols described here were carried out in accordance with the declaration of Helsinki.

### Experimental design

A case-control and ecological experimental design was used for this investigation. Once the participants had finished their intervention in the investigation (see experimental protocol below), they were divided into two groups according to their serum CK concentration after the marathon race, as follows: (1) low CK responders with serum CK concentration < 400 U·L^-1^; (2) high CK responders with serum CK concentration ≥ 400 U·L^-1^. This cutoff point for serum CK concentration was based on a previous investigation that suggested this value as the break point for CK release after endurance exercise [6, 27]. Besides, average serum CK concentration of previous investigations in this same marathon and with participants of the same characteristics (e.g., amateur marathoners) was ~445 U·L^-1^ [7, 28] which suggests the adequacy of the cutoff point to identify low and high CK responders.

### Genotypes

The polygenic analysis performed in the current investigation was inspired by the investigation carried out by Ruiz, Gomez-Gallego [29], which aimed to identify the optimum polygenic profile for endurance exercise. However, in this case we have analyzed seven genetic polymorphisms that are candidates to explain individual variations in exercise-induced muscle damage, specifically CK response to muscle-damaging exercise. The candidate genes were selected from the analysis performed by Yamin, Meckel [30] for the relationship between genetics and rhabdomyolysis, and they coincide with most of the genes associated with exercise-induced muscle damage suggested in a recent review [31] published after this investigation was finished. The seven genetic polymorphisms included in the current analysis were as follows (Table 2):

- The 287bp Ins/Del polymorphism (rs4340) of the ACE gene (location: 17q23.3)
- The p.R577X polymorphism (rs1815739) of the ACTN3 gene (location: 11q13.1)
- The NcoI polymorphism (rs1803285) of the CKMM gene (location: 13q13.32)
- The C13790G polymorphism (rs3213221) of the IGF2 gene (location: 11p15.5)
- The 174G>C polymorphism (rs1800795) of the IL6 gene (location: 7p15.3)
- The C37885A polymorphism (rs28497577) of the MLCK gene (location: 3q21.1)
- The 308G>A polymorphism (rs1800629) of the TNFα gene (location: 6p21.33)

**Table 2:** Studied polymorphisms in amateur marathon runners. Genotype scores have been based on previous research as follows: 2 = optimal genotype; 1 = standard genotype; 0 = suboptimal genotype for muscle damage during exercise.

Symbol	Gene	Polymorphism	dbSNP	Genotype score	Frequency	References
ACE	Angiotensin I converting enzyme	Alu 287bp (I/D)	rs4340	0 = II	14.9%	[12]
				1 = ID	32.8%	
				2 = DD	52.2%	
ACTN3	Actinin, alpha-3	c.1858C>T; p.R577X	rs1815739	0 = XX	10.4%	[10, 14, 16]
				1 = RX	61.2%	
				2 = RR	28.4%	
CKMM	Creatine kinase, muscle type	c.800A>G (NcoI)	rs1803285	0 = AA	58.2%	[5]
				1 = AG	41.8%	
				2 = GG	0.0%	
IGF2	Insulin-like growth factor II	c.-6-285G>C (C13790G)	rs3213221	0 = GG	16.4%	[18]
				1 = GC	38.8%	
				2 = CC	44.8%	
IL6	Interleukin 6	c.-174G>C	rs1800795	0 = CC	44.8%	[19, 20]
				1 = GC	41.8%	
				2 = GG	13.4%	
MLCK	Myosin light chain kinase	c.62C>A; p.P21H (C37885A)	rs28497577	0 = CC	88.1%	[23]
				1 = CA	11.9%	
				2 = AA	0.0%	
TNFα	Tumor necrosis factor	c.-308G>A	rs1800629	0 = GG	77.6%	[19, 20]
				1 = GA	22.4%	
				2 = AA	0.0%	

Genotyping methods have been described elsewhere [23] and were in accordance with rigorous recommendations for replicating human genotype-phenotype association investigations. Briefly, genomic DNA was isolated from the whole blood obtained before the race (QIAamp^®^ DNA Blood Mini Kit, QIAGEN, The Netherlands) according to the manufacturer’s protocol and the genotyping was performed using a TaqMan^®^ SNP genotyping assay (Life Technologies^™^, USA) that employs the 5’ nuclease activity of Taq DNA polymerase to detect a fluorescent reporter signal generated during Real-Time PCR reactions. Amplification and detection were performed using a real-time PCR system (Applied Biosystems^®^ Steponeplus^™^ Real-time PCR system, Life Technologies^™^, USA). All these analyses were performed in the certified EuroEspes Laboratory (A Coruña, Spain).

### Total genotype score

The combined influence of all the seven studied polymorphism was computed following the procedure by Williams and Folland [32]. Initially, we allocated scores to each genotype within a particular polymorphism. Typically, the candidate polymorphisms were bi-allelic, providing three possible genotypes. The homozygote genotype associated with a reduced CK response after exercise or reduced signs of exercise-induced muscle damage (e.g., leakage of other intramuscular proteins, muscle fatigue, muscle pain, etc) was given a score of 2 points, with a linear trend applied (e.g., heterozygotes were scored 1 point and the other homozygotes were scored 0 points). All the genetic polymorphism received the same score because there is no objective information to determine the prominence among these polymorphisms for reduced CK response or reduced muscle damage. Then, the scores obtained in each genetic polymorphism were summed for a perfect total genotype score of 14 point that represents the optimal genotype to resist exertional muscle damage/low CK release after exercise. In contrast, a total genotype score of 0 points represents the worst possible genotype to resist exertional muscle damage/high CK release after exercise. The scores included in this investigation can only be interpreted within the context of this paper because other polymorphisms may be associated with exertional muscle damage in the future [31]. Finally, the distribution of the total genotype scores was compared between the low CK responders and high CK responders identified in this investigation. The scores allocated to each genotype and the references that support this analysis can be viewed in Table 2. A more detailed explanation for each genotype score can be obtained from the introduction section.

### Experimental protocol

All the participants in this research underwent the same testing under the same experimental conditions. Participants were encouraged to refrain from pain-relieving strategies (e.g., analgesic medications, massage or ice application), vigorous exercise, caffeine and alcohol during the 48-h prior to the race and fulfillment was verified by individual diaries. A venous blood sample was obtained 24-h before the onset of the competition following a 10-min resting period seated in a chair. Then, participants performed a 10-min warming-up protocol and performed two maximal countermovement vertical jumps on a force platform (Quattrojump, Kistler, Switzerland), as previously described [2].

The day of the race, participants had their habitual pre-competition meal -at least 3 h before the onset of the race- and they were instructed to ingest 500 mL of tap water -2 h before the start of the race-, as previously described [33]. All participants competed in the 2015 edition of the Rock’n’Roll Madrid Marathon with no information about pacing or nutritional strategies to be held during the competition. The race was held in April with a mean dry temperature of 13.0 ± 1.0°C (range from 11 to 14°C, temperature readings at 30-min intervals from 0- to 5-h after the race onset) and a mean relative humidity of 88 ± 1% (range from 88 to 89%). For each participant, the time taken from the start line of the race to the finish line (net time) was calculated by means of an individual timing-chip. All these standardizations were set to minimize the influence of the research during the competition.

Within 2 min of the conclusion of the race, the runners went to a research area located next to the finish line and they repeated the two countermovement vertical jumps, replicating the protocol performed before the race. At this time, the rating of perceived exertion was assessed using the Borg scale (from 6 to 20 arbitrary units, A.U.). Participants then rested for 5 min seated in a chair and a venous blood sample was obtained. During this time, lower-limb muscle pain at the end of the race was measured using a 10-cm visual analog scale, as previously described [17].

### Blood samples

A portion of each blood sample was introduced *in situ* into a blood glucose analyzer (Accu-chek, Roche, Spain) to determine blood glucose concentration. The whole blood was also analyzed for hemoglobin concentration (Coulter ACT5 Diff CP; Beckman-Coulter Instruments, Villepinte, France) and hematocrit was measured by triplicate using microcentrifugation. Afterwards, the blood samples were allowed to clot and they were centrifuged at 5000 g for 10 min to obtain serum samples. The serum samples were introduced in clean tubes and frozen at -80°C until they were analyzed for creatine kinase and myoglobin concentrations by means of an autoanalyzer (Access II, Beckman-Coulter Instruments, USA) in a latter date. Serum electrolyte concentrations (Na^+^, Cl^-^ and K^+^) were also determined in all serum samples (Spotlyte, Menarini Diagnostics, Madrid, Spain).

### Statistical analysis

The data were analyzed with the statistical package SPSS version 19.0 (SPSS Inc., Chicago, IL). The significance level was set at *P* < 0.05. Data are presented as mean ± SD or as frequencies for each group of participants. In a previous analysis, the normality of each variable was tested with the Kolmogorov-Smirnov test. Post-race creatine kinase and myoglobin concentration (and their pre-to-post race changes) were the only variables that did not follow a normal distribution and thus they were analyzed with non- parametric statistics. The comparison between groups (low CK responders *vs*. high CK responders) was performed using Student’s t test for independent samples or using a two-way ANOVA (group × time). For the non-parametric variables, the U-Mann-Whitney test was used for comparison between groups. For the between-group comparison of the individual score of each gen, crosstab with Somers’ d statistic was calculated with Bonferroni adjustment for multiple testing (*P* < 0.007). The effect size (ES) was calculated in all pairwise comparisons and the magnitude of the effect size was interpreted using Cohen’s scale [34].

## Results

**Fig 1:**
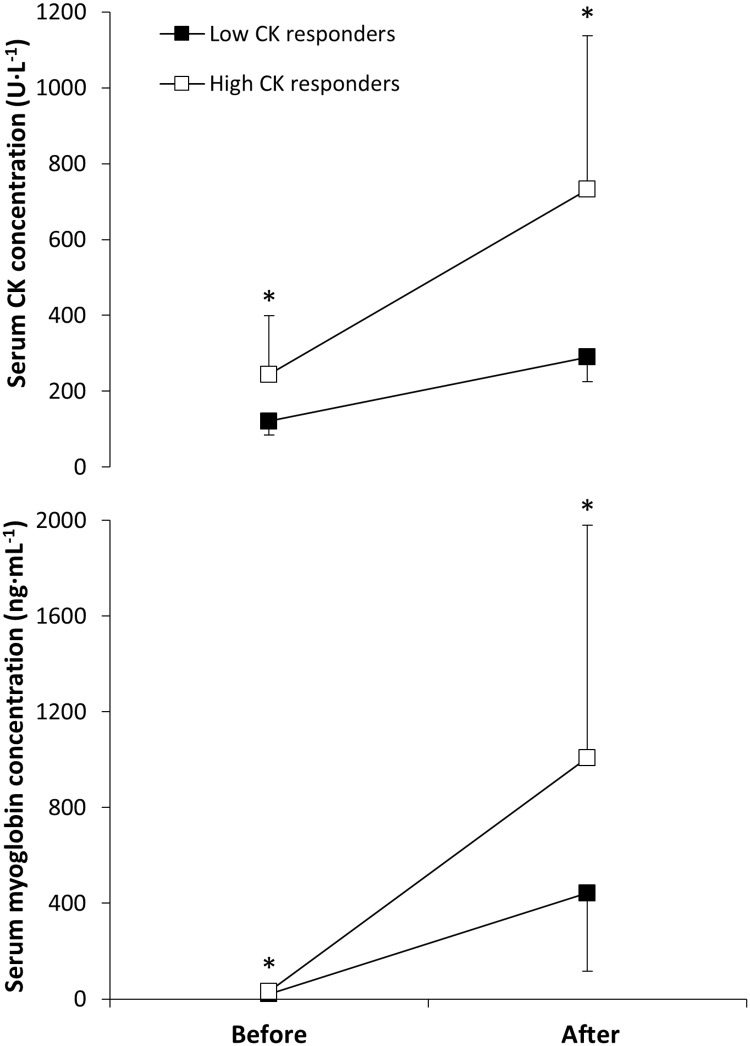
Serum Creatine Kinase (CK) and myoglobin concentrations before and after a marathon competition in low and high CK responders. Data are mean ± SD. (*) Different from low CK responders at *P* < 0.01.

The distribution of each genotype polymorphism for the whole-group is presented in Table 2. All the genotypes were represented in the study sample except GG homozygosity for the CKMM NcoI, AA homozygosity for the MLCK C37885A and for AA homozygosity for the TNFα 308G>A. Low CK responders had lower values of serum CK concentration before (ES = 3.35; *P* < 0.01) and after the race (ES = 6.86; *P* < 0.01, Fig 1) than high CK responders. Similarly, low CK responders had lower values of serum myoglobin concentration before (ES = 1.65; *P* < 0.01) and after the race (ES = 1.72; *P* < 0.01, Fig 1) than high CK responders. The pre-to-post-race change in serum CK and myoglobin concentration was also smaller in low CK responders *vs*. high CK responders (Table 3).

**Table 3:** Pre-to-post-race changes in serum CK, myoglobin concentration and jump height, race time and self-reported fatigue and muscle pain of runners with low and high CK responses after a marathon. Data are mean ± SD.

Variable	Low CK responders	High CK responders	Δ	ES	P value
Serum CK concentration change (%)	253 ± 79	339 ± 142	34.0%	1.09	<0.01
Serum myoglobin concentration change (%)	2248 ± 1822	3302 ± 3073	46.9%	0.58	0.04
Race time (min)	226 ± 33	231 ± 34	2.5%	0.17	0.50
Jump height change (%)	-40.6 ± 18.0	-40.6 ± 19.4	0.1%	0.16	0.60
Leg muscle power change (%)	-32.9 ± 14.6	-30.6 ± 18.2	7.0%	0.16	0.60
Borg-scale (A.U.)	15.3 ± 1.9	15.5 ± 2.0	0.8%	0.06	0.82
Self-reported leg muscle pain (cm)	6.34 ± 1.59	6.14 ± 1.80	-3.2%	0.13	0.65

**Fig 2:**
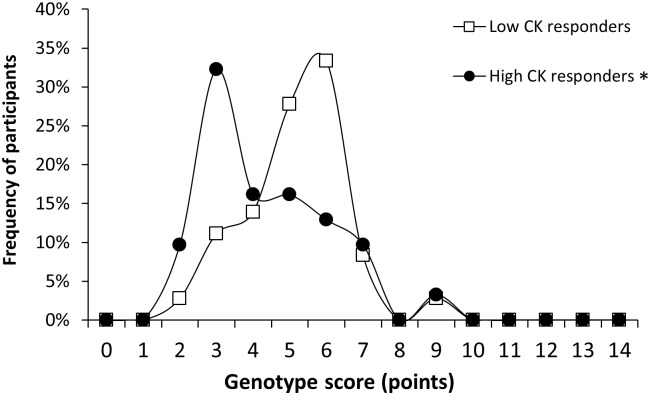
Frequency distribution of genotype scores (0–14 point) in low and high CK responders during a marathon competition. (*) Different from low CK responders at *P* < 0.05.

**Fig 3:**
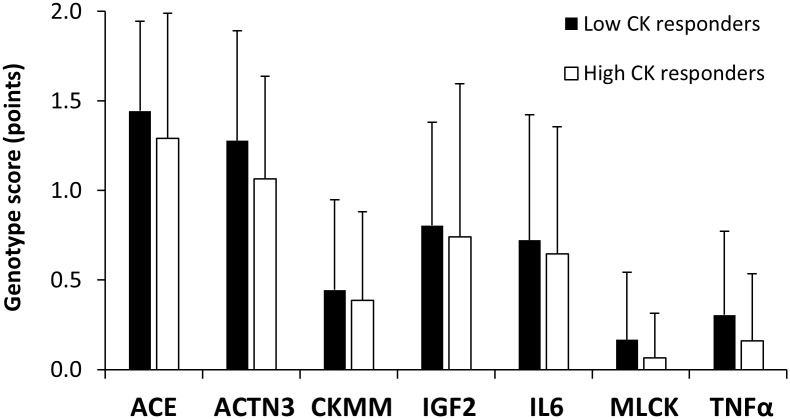
Individual genotype scores in low and high CK responders during a marathon competition. Data are mean ± SD.

Although there were no between-group differences in age, anthropometric characteristics, best race time in the marathon, running experience and training habits (Table 1), the distribution of the total genotype score was significantly different between low CK *vs*. high CK responders (*P* = 0.03, Fig 2). In fact, low CK responders had a higher mean (5.2 ± 1.4 *vs*. 4.4 ± 1.7 point, Δ = -15.7%, ES = 0.58; *P* = 0.04), median (5 *vs*. 4 points) and mode (5 *vs*. 3 points) for total genotype score than high CK responders. The differences in the distribution of total genotype score were based on the summed influence of all seven genotype polymorphisms, although there were no significant differences between low CK responders *vs* high CK responders for the separate scores of each gen (Fig 3).

Total race time was very similar between groups (Table 3). From similar pre-race values (25.2 ± 3.9 *vs*. 25.6 ± 4.2 cm, Δ = 1.6%, ES = 0.10; *P* = 0.71), low CK responders and high CK responders attained similar jump height reductions (Table 3). The same pattern was found for pre-to-post-race leg muscle power change in the two groups of marathoners. Self-reported exertion and leg muscle pain after the race were also very similar in low CK *vs*. high CK responders (Table 3). Pre-race, post-race and changes within the race in body mass, blood glucose concentration, serum electrolyte concentration and blood and plasma volume were very similar between groups (all ES <0.4 and *P* > 0.05).

## Discussion

The aim of the current research was to investigate the summed effect of seven SNPs of candidate genes (ACE, ACTN3, CKMM, IGF2, IL6, MLCK, TNFα) on the levels of serum CK concentration and other signs of exercise-induced muscle damage attained after a real marathon competition. For this purpose, we used the model proposed by Williams and Folland [32] to assign to each marathoner a total genotype score derived from the accumulated combination of these seven SNPs (from 0 to 14 points). Besides, we used the serum CK concentration after the race as the differential outcome to classify marathoners, because this variable has been the most used outcome in previous investigations on this topic [10, 12, 14, 16, 18–20]. The main outcomes of this investigation were: (a) marathoners with the lowest serum CK concentration after the race (on average 290 ± 65 U·L^-1^) presented a significantly higher value and different distribution (Fig 2) for total genotype scores than marathoners with the highest serum CK response (on average 733 ± 405 U·L^-1^); (b) the higher genotype score in low CK responders was the result of the combined effect of the seven SNPs analyzed because each individual genotype score tended to be higher in this group of runners (Fig 3); (c) the two groups of marathoners had no differences in age, anthropometric characteristics, running experience and training habits (Table 1), competed in the race with a very similar running pace and presented similar values of muscle fatigue and self-reported muscle pain (Table 3). All this information suggests that the genotypes related to lower scores in the investigated SNPs (Table 2) result in a higher leakage of intramuscular components into the blood circulation despite similar values of training, exercise intensity and muscle pain. Since this phenotype can be strongly related to the severity of exertional rhabdomyolysis, especially to renal failure in marathoners [4, 25], having a favorable polygenic profile for exertional-induced muscle damage may reduce the likelihood of suffering fatal exertional rhabdomyolysis in the marathon.

The current investigation has been possible thanks to the results of previous research on this topic (Table 2) and thus, we want to acknowledge the labor of different research groups to determine the influence of different SNPs on the levels of exertional rhabdomyolysis. However, this is a novel report because it includes actual data on the polygenic profile of marathoners with the same ethnic origin who have different CK responses. While previous reviews have contemplated the inclusion of one or two SNPs to explain the differences in CK response and other signs of exertional rhabdomyolysis [30, 31], this is the first investigation that includes the expected interrelationship of seven candidate genes for a unique phenotype (serum CK concentration after a marathon). A second strength of this experimental design is the use of a real competitive marathon as the exercise scenario to develop muscle damage. While most of the previous investigations have used well-controlled eccentric exercise of the elbow, we believe that the results of the current investigation are more applicable to athletes since the marathon is a more common exercise discipline.

An excellent and thoughtful explanation of the mechanistic associations of the seven investigated SNPs with a lower capacity of skeletal muscle to resist strain can be found elsewhere [31]. Briefly, some of the genes such as the ACTN3 and, MLCK and CKMM are directly related with the muscle fiber properties to produce force and to obtain energy. In the case of the ACTN3 R577X, the XX genotype is associated with a premature stop codon that leads to the production of a non-functional α-actinin-3 in type II fibers [14]. Although this genotype is compensated by metabolic adaptations towards a more oxidative and more efficient metabolism in type II muscle fibers [15], and increased gene expression of sarcomeric z-disk proteins [35], the lack of α-actinin-3 has been related to a lower capacity to resist intense/repeated muscle contractions [10, 14, 16, 17]. Thus, it is likely that this mechanism contributed to the higher levels of serum CK concentration found in high CK responders with the ACTN3 XX genotype. Regarding MLCK C37885A, Childers and McDonald [21] incubated skinned type II fibers with MLCK in rat psoas muscle and found a significant increase in Ca^2+^ sensitivity that ultimately produced greater force and power. In this respect, Clarkson, Hoffman [22] suggested that this SNP may be related to exertional rhabdomyolysis due to enhanced regulatory light chain phosphorylation leading to higher muscle strain. In the case of CKMM NcoI, this SNP is mapped to the 3′ untranslated region, which means it could affect the localization, translation efficiency and stability of the mRNA, which might mediate the location and function of the protein [31]. However, more information is necessary to improve the understanding of the influence of CKMM NcoI SNP on exertional rhabdomyolysis.

ACE and TNF genes and their respective SNPs might influence exertional rhabdomyolysis levels by different pathways related to inflammatory processes, exercise-induced muscle hypertrophy and muscle regeneration following exercise or muscle injury [31, 36]. In addition, varying IGF-II levels potentially caused by the IGF2 C13790G could modulate satellite cell activation and differentiation thus affecting the properties of trained skeletal muscle against high-intensity concentric and eccentric contractions [37]. The IL6 174G>C may be related to the pro-inflammatory properties of skeletal muscles and the modulation of the release of different cytokines such as TNF and might play an important role in satellite cell proliferation after intense exercise [38]. The sum of several of these proposed and suggested mechanisms due to favorable SNPs might be responsible for the ameliorated serum CK concentration after the race, because a higher total genotype score is likely related to a higher engagement of these protective mechanisms.

As indicated above, we used serum CK concentration as the grouping variable to improve the comparison of our data with previous investigations since this variable was the preferred one in most investigations and it is the most sensitive laboratory indicator of myocyte injury [39]. However, the increases in serum CK have no toxic effects [40, 41]. On the other hand, serum myoglobin concentration is a more important variable in terms of clinical significance for exertional rhabdomyolysis since the serum increases of this intramuscular protein may cause renal tubular obstruction, direct nephrotoxicity, intrarenal vasoconstriction and ultimately acute kidney injury [42]. Briefly, myoglobin cannot be reabsorbed when it is present in excessive amounts in the renal tubules while vasoconstriction and hypovolemia–exacerbated by exercise, especially in hot environments- result in water reabsorption in renal tubules which in turn increases further myoglobin concentration in the glomeruli. The latter produces the formation of casts that obstruct renal tubules while the most common sign of exertional rhabdomyolysis is presence of urine with a brown-red color.

In the current investigation, low CK responders increased 2248 ± 1822 times their pre-competition serum myoglobin concentration while high CK responders increased by 3302 ± 3073 times their pre-race serum myoglobin content, even when high CK responders had already a higher serum myoglobin concentration at the beginning of the race. In the case of using post-race serum myoglobin concentration as the grouping variable, the total genotype score would also be different between low and high myoglobin responders (5.2±1.7 vs. 4.4±1.5 point, *P* = 0.02) which confirms the importance of having an advantageous genotype combination to reduce the probability of acute renal failure as the result of exertion rhabdomyolysis.

Aside from its strengths, the present investigation also presents some limitations derived from the experimental design selected. Because this study was carried out on a real competitive marathon, factors such as age, anthropometry, pre-competition diet, training volume and running pace during the race were not controlled. Nevertheless, we registered this information and based on the absence of between-group differences (Table 1) we believe that these factors had a negligible influence on the outcomes of this investigation. It is well established that some of the symptoms of exertional rhabdomyolysis are more present 24-to-48 h after the cessation of the exercise activity [4] but our experiment only extended to the end of the race. It would be necessary to investigate whether the influence of genotype profile is also present during the recovery phase of exercise-induced muscle damage. Lastly, this investigation has included seven genotype polymorphisms while other genes such as the chemokine ligand 2 (CCL2) and its receptor chemokine receptor 2 (CCR2; [43]), the LPIN1 [44], the IL1B gene and/or the osteopontin gene [31] have also been identified as candidate genes for an elevated CK response and an exaggerated damage response to exercise. Even, DNA telomere lengths can be associated to the level of exercise-induced muscle damage [45]. Thus, these and other genetic variants that are likely to appear in the foreseeable future can also help to explain variations in CK response.

In summary, marathoners with a lower CK response after a marathon had a more favorable polygenic profile than marathoners with high serum CK concentrations. Besides, low CK responders showed significantly lower serum myoglobin concentrations after the race than their high CK counterparts. This suggests a decisive role of the seven genetic polymorphisms included in this investigation on the leakage of intramuscular proteins into the blood stream during muscle-damaging exercise that ultimately can affect the development of myoglobin-induced renal failure [3]. Thus, individuals with a lower total genotype score, because of a suboptimal polygenic profile, could be more prone to suffering exertional rhabdomyolysis in exercises such as the marathon. Yet other SNPs, in addition to exercise training, environmental conditions, dietary supplements and medications [10], might also play a role in the values of CK and the probability of developing exertional rhabdomyolysis during endurance exercise.

## Abbreviations

ACE: Angiotensin I converting enzyme
ACTN3: actinin, alpha-3
CK: creatine kinase
CKBB: creatine kinase, brain type
CKMB: creatine kinase, cardiac muscle type
CKMM: creatine kinase, skeletal muscle type
IGF2: insulin-like growth factor II
IL6: interleukin 6
MLCK: myosin light chain kinase
SNP: single nucleotide polymorphism
TNFα: tumour necrosis factor
